# An ultralow dose of the NADPH oxidase inhibitor diphenyleneiodonium (DPI) is an economical and effective therapeutic agent for the treatment of colitis-associated colorectal cancer

**DOI:** 10.7150/thno.43938

**Published:** 2020-05-20

**Authors:** Yue Kuai, Hao Liu, Dongyu Liu, Yunlong Liu, Ye Sun, Jiansheng Xie, Jiachun Sun, Yong Fang, Hongming Pan, Weidong Han

**Affiliations:** 1 Department of Medical Oncology, Sir Run Run Shaw Hospital, College of Medicine, Zhejiang University, Hangzhou, Zhejiang, China.; 2 Laboratory of Cancer Biology, Institute of Clinical Science, Sir Run Run Shaw Hospital, College of Medicine, Zhejiang University, Hangzhou, Zhejiang, China.; 3 Department of Orthopedics, the Second Affiliated Hospital, Zhejiang University School of Medicine, Hangzhou, China.; 4 The First Affiliated Hospital, College of Clinical Medicine of Henan University of Science and Technology.

**Keywords:** colitis-associated colorectal cancer, diphenyleneiodonium, NADPH oxidase inhibitor, macrophage, polarization

## Abstract

Long-term inflammatory stimulation is considered one of the most important causes of colorectal cancer. Diphenyleneiodonium (DPI), an NADPH oxidase inhibitor, can inhibit a variety of inflammatory responses. However, the systemic toxicity of DPI limits its clinical application. Whether DPI can inhibit colitis-associated colorectal cancer (CAC) at ultralow concentrations remains unknown.

**Methods**: CAC was induced by azoxymethane (AOM) injection followed by treatment with dextran sulfate sodium (DSS), and DPI was intraperitoneally injected (i.p.) in the first cycle for 21 days. Colon tissue was collected and analyzed by western blotting. Immune cell infiltration and macrophage polarization were examined by immunohistochemistry, immunofluorescence, or real-time polymerase-chain reaction (PCR). Reactive oxygen species (ROS) production was measured by flow cytometry.

**Results**: Ultralow dose DPI significantly ameliorated the DSS-induced colitis and attenuated the colon tumorigenesis in the mouse model of AOM/ DSS-induced CAC. Mechanistically, an ultralow dose of DPI inhibited the production of pro-inflammatory cytokines, (tumor necrosis factor (TNF)-α and interleukin (IL)-6), reduced the macrophage infiltration and classical polarization, and induced the ROS generation. These effects were found to be related to the inhibition of the phosphorylation of signal transducer and activator of transcription 3 (STAT3), mitogen-activated protein kinase (MAPK), and nuclear factor kappa B (NF -κB).

**Conclusion**: The present study revealed that an ultralow dose of DPI, with no significant systemic toxicity involved, may be an effective way to prevent the occurrence and development of CAC.

## Introduction

Colitis-associated colorectal cancer (CAC) is a multistep and multifactorial malignancy with a poor response to treatment and high mortality 1. In general, the development of CAC is closely related to chronic inflammation, and studies have shown that patients with inflammatory bowel disease (IBD), including ulcerative colitis (UC) and Crohn's disease (CD), are at an increased risk of developing colorectal cancer (CRC) 2. In addition, both the sporadic and heritable colorectal cancers are also associated with inflammation 3. Epidemiological studies have demonstrated that the anti-inflammatory therapies can effectively reduce the incidence of colorectal cancer in patients with IBD 4,5. Thus, the inhibition of inflammation is a potential strategy to prevent the development of CAC.

The inflammatory tumor microenvironment, which is composed of fibroblasts, mesenchymal cells, and innate and adaptive immune cells, plays an important role in tumor progression 6. Among the immune cells, macrophages play major roles in inflammation-related cancer and display considerable plasticity and heterogeneity depending on the microenvironmental stimuli 7. Classically activated M1 macrophages are induced by interferon (IFN)-γ and bacterial lipopolysaccharide (LPS) to secrete a wide range of proinflammatory cytokines, such as interleukin (IL)-6, IL-1β, IFN-γ, and inducible nitric oxide synthase (iNOS), leading to tissue damage 8,9. In contrast, alternatively activated M2 macrophages, which are induced by IL-4 and IL-13, contribute to the resolution of colitis via the upregulation of arginase (Arg)-1, mannose receptor (MR)-1, chitinase-like 3 (Chil3/Ym1), IL-10, and Fizz1, all of which promote tissue repair 10. Macrophages play contrasting roles in cancer progression depending on their polarization. Ong et al. reported that macrophages are predominantly polarized toward a classically activated M1 phenotype and express pro-inflammatory cytokines, such as IFN-γ, which can activate cytotoxic CD8^+^ T-cells to inhibit tumor growth 11. Several other studies have confirmed that M2 macrophages promote tumor growth by facilitating angiogenesis, immunosuppression, and remodeling of extracellular matrix 12,13. For example, the p38/MAPK-activated protein kinase 2 (MK2) pathway promotes the development of colonic tumors by regulating the polarization state of the tumor-promoting M2 macrophages to modulate the tumor microenvironment and enhance tumor angiogenesis 14. Altogether, these findings suggest that there is a delicate balance between the tumoricidal and tumor-promoting functions of macrophages.

Increasing evidence has indicated the critical role of nicotinamide adenine dinucleotide phosphate oxidases (NOX), which catalyzes the generation of reactive oxygen species (ROS), such as superoxide and hydrogen peroxide from molecular oxygen, in the pathogenesis of gastrointestinal inflammation 15,16. NOX2 is the primary superoxide-producing enzyme in macrophages and is closely associated with the M1 function 17. Once NOX2 is activated, superoxide can interact with nitric oxide to form peroxynitrite or be dismutated to form hydrogen peroxide, which can contribute to the pathogen killing, oxidation and nitration of proteins and lipids, and exacerbation of the inflammatory disease 15,18. Diphenyleneiodonium (DPI) is a widely used NOX2 inhibitor that interacts with the catalytic subunit of NOX2, leading to the formation of relatively stable covalent adducts 16. Unfortunately, its high toxicity and lack of specificity at standard micromolar concentrations have limited its clinical and experimental applications. Wang et al. demonstrated that at subpicomolar concentrations, DPI (10^-13^ M or 10^-14^ M) specifically inhibited the activation of NOX2 and subsequently protected the dopaminergic neurons against xenobiotics-induced toxicity 19. Moreover, they reported that DPI at an extremely low dose (10 ng/kg/day, subcutaneously administered via a 2-week infusion) effectively protected the dopaminergic neurons after the loss of ~30% of the dopaminergic neurons in two mouse models of Parkinson's disease, the inflammation-driven and subchronic 1-methyl-4-phenyl-1,2,3,6-tetrahydropyridine (MPTP) models 20. These findings indicate that DPI at ultralow doses can be used as a potential treatment for animal and clinical studies in future. However, whether DPI at ultralow doses has protective functions in CAC needs to be determined.

In this study, we investigated the functional importance of an ultralow dose of DPI in suppressing tumorigenesis during the progression of CAC. We have demonstrated that an ultralow dose of DPI can inhibit the intestinal inflammatory response by reducing the macrophage recruitment and M1 macrophage activation. These results indicate that an ultralow dose of DPI may serve as a promising treatment for the prevention of CRC progression in IBD patients.

## Methods

### Mouse and animal models

C57BL/6 mice (male, 6-8 weeks old, 20-25 g, Shanghai Institute of Material Medicine, Chinese Academy of Science, China) were housed in a pathogen-free facility and were used in accordance with the protocols approved by the Animal Care and Use Committee of Zhejiang University.

For the induction of acute colitis, 6-8 weeks old mice were randomly divided into four groups: (a) water only (normal), (b) receiving DPI for 15 days (DPI), (c) receiving DSS for 6 days and phosphate-buffered saline (PBS) for 15 days (DSS+PBS), and (d) receiving DSS for 6 days and DPI for 15 days (DSS+DPI). Mice were administered 2% (w/v) DSS (MW, 36,000-50,000; MP Biomedicals, Santa Ana, CA, USA) in sterile drinking water for 6 days. DPI (10 ng/kg, #S8639, Selleck Chemicals, Houston, USA) or PBS was administered by intraperitoneal injection (i.p.) once daily according to the time schedule. Mice were monitored daily to determine their body weight and were checked for diarrhea and bleeding, which were scored as previously described 21.

The induction of CAC followed a previously reported method 22. Briefly, mice received a single intraperitoneal injection of 10 mg/kg azoxymethane (AOM) (#A5486, Sigma-Aldrich, MO, USA) and were maintained on a regular diet and drinking water for 7 days. The mice were subjected to 3 cycles of DSS administration (1.8%, w/v) for 6 days, with a 15-day recovery period. DPI or vehicle control (PBS) was intraperitoneally injected at 10 ng/kg body weight/day every day in the first cycle for 21 days of treatment. The mice were euthanized on day 80, and the colon was removed and washed with PBS before analysis.

For the induction of systemic endotoxemia, 7-8 week old mice were intraperitoneally injected with 5 mg/kg LPS (#L4130, Sigma-Aldrich). Mice were then injected intraperitoneally with 10 ng/kg DPI within half an hour. Four hours later, blood samples were collected by cardiac puncture under isoflurane anesthesia. Blood samples were stored at 37 °C for 4 h, and then serum was separated by centrifugation at 5,000 × *g* for 10 min.

To establish the peritonitis model, 7-8 weeks old mice were intraperitoneally injected with 2 mL of thioglycolic acid (4%, w/v), and then the mice were injected intraperitoneally with 10 ng/kg DPI or PBS once daily for 4 days. At day 4, after the thioglycolate administration, mice were euthanized, and the peritoneal cavity was flushed with PBS to harvest the cells. The collected peritoneal cells were counted and used for phenotypic analysis by flow cytometry.

### Mouse tissue processing

The intestines were immediately removed and flushed with cold PBS, and the distance between the ileocecal junction and proximal rectum was measured. After splaying the colon along its length, the number and size of the tumors were quantified. The colons were divided into several sections and were either fixed in 10% neutral-buffered formalin (Sigma-Aldrich) or used for western blotting, RNA extraction, and isolation of intestinal epithelial cells.

### Histopathology and immunostaining

For histological evaluation, the colons were fixed in 10% neutral-buffered formalin overnight at 4 °C, embedded in paraffin, and sectioned. Samples were stained with hematoxylin and eosin (H&E)(Huabio, Hangzhou, China).

For the immunohistochemical (IHC) staining, the tissue slides were deparaffinized with xylene (Aladdin, Shanghai, China) and rehydrated with ethanol (Ante, Suzhou, China). After inhibiting the endogenous peroxidase using 3% H_2_O_2_ in methanol (Lingfeng, Shanghai, China), the sections were rinsed with PBS and the slides were blocked with 10% bovine serum albumin (BSA) (Meilunbio, Dalian, China) for 1 h at 20-25 °C, and were then incubated with primary antibodies (listed in Table 1) overnight at 4 °C and then the secondary antibodies at room temperature for 30 min. Following incubation, the reaction products were visualized with diaminobenzidine (Maxim, Fuzhou, China) as a chromogen and counterstained with hematoxylin.

For the immunofluorescence (IF) staining, the tissue slides were deparaffinized with xylene and rehydrated with ethanol. After inhibiting the endogenous peroxidase by 3% H_2_O_2_ in methanol, the sections were rinsed with PBS and the slides were blocked with 10% BSA for 1 h at room temperature, and then incubated with the primary antibodies (listed in Table 1) overnight at 4 °C, followed by incubation with the secondary antibodies at room temperature for 30 min. The reaction products were visualized by incubation with 4´,6-diamidino-2-phenylindole (DAPI; #MA0128, Meilunbio) as a chromogen. Signals were detected with Alexa Fluor 488 (Arg1) and Alexa Fluor 594 (iNOS).

### Goblet cell assay

To identify the goblet cells, tissue slides were stained with periodic acid-Schiff (PAS) (#G1281, Solarbio, Beijing,China) according to the manufacturer's instructions. Goblet cells appeared bluish violet after PAS staining. Counterstaining with hematoxylin was used to identify the nuclei.

### Isolation of the intestinal epithelial cells and colonic lamina propria from mouse colon

For the isolation of intestinal epithelial cells (IECs), fresh intestinal tissues were harvested from sacrificed mice as mentioned above and flushed with ice-cold PBS. The colon segments were opened longitudinally, cut into 2 mm pieces, and incubated in 30 mM ethylenediaminetetraacetic acid (EDTA, Sigma-Aldrich) and 1.5 mM dithiothreitol (DTT, Sigma-Aldrich) in Hank's balanced salt solution (HBSS, Sigma-Aldrich) at 4 °C for 30 min to remove mucus. Then, the mucosa was washed in PBS and incubated in pre-warmed HBSS with 30 mM EDTA and 2% fetal bovine serum (FBS) for 90 min in a 37 °C shaker. The supernatant (IEC and tumor cells) was collected and washed twice with cold PBS.

The colonic lamina propria cells were isolated from the tissue remaining after the DTT and EDTA treatments. The tissues were incubated for 1 h in a 37 °C shaker and digested in 0.3 U/mL collagenase D (Roche, Basel, Switzerland) in Roswell park memorial institute (RPMI) media. The samples were filtered through a 40 μm cell strainer (Corning, NY,USA), washed with PBS, and resuspended in 6 mL of 40% Percoll (Sigma-Aldrich). Approximately 2 mL of 70% Percoll was carefully added to the bottom of 40% Percoll cell suspension using a long glass micropipette. The samples were centrifuged at 2400 rpm for 30 min, and cells from the 40-70% Percoll interface were transferred to fresh RPMI media. The cells were collected by centrifugation at 1300 rpm for 10 min at 4 °C.

### Fluorescein isothiocyanate-dextran assay

Mice were administered 0.6 mg/kg fluorescein isothiocyanate (FITC)-labeled dextran (molecular weight, 4,000; Sigma-Aldrich) in PBS by oral gavage. Four hours later, blood samples were collected by cardiac puncture, and the fluorescence was measured using a Synergy H4 Hybrid Reader (Biotek, VT, USA).

### Cell culture

RAW264.7, MC38, and NCM460 cell lines were purchased from the Cell Bank of the Chinese Academy of Science (Shanghai, China) and cultured in Dulbecco's modified eagle medium (DMEM) (Geneodx, Shanghai,China) containing 10% heat-inactivated FBS (HyClone, UT, USA), 100 U/mL penicillin, and 100 μg/mL streptomycin at 37 °C in 5% CO2. The THP-1 human monocytic cell line was provided by Dr. Jia He 23 and was cultured in RPMI 1640 medium. THP-1 monocytes differentiated into macrophages on treatment with 100 ng/mL Phorbol 12-myristate (PMA, #S1819, Beyotime, Shanghai, China) for 24 h. For the bone marrow-derived macrophage (BMDM) culture, bone marrow was flushed from mouse femurs and tibias and cultured in DMEM supplemented with macrophage colony-stimulating factor (20 ng/ml, PeproTech, HJ, USA) and 10% heat-inactivated FBS.

### Cytokine assay

The cytokine levels of IL-6, tumor necrosis factor (TNF)-α, and monocyte chemoattractant protein-1 (MCP-1) in the cell culture supernatants and serum from experimental mice were detected using commercial enzyme-linked immunosorbent assay (ELISA) kits (Invitrogen, CA, USA) according to the manufacturer's instructions. The results were calculated in pg/mL.

### Flow cytometric analysis

Flow cytometric analysis of macrophages was performed by staining of the cell surface markers. Cells (1-2×10^6^) were stained in 1 mL PBS with the Live/Dead-Aqua according to manufacturer's instructions (#L34957, Invitrogen), washed in an additional 2 mL PBS by centrifugation at 1000 rpm for 5 min, and then resuspended in 100 μL PBS containing 2% FBS. The following fluorochrome-conjugated anti-mouse antibodies were added at manufacturer's recommended concentrations for 30 min at room temperature: CD45-APC (#17-0451-82e, Bioscience, Shanghai, China), CD11b-Perp/Cy5.5 (#550993, Becton, Dickinson and Company, NJ, USA), and F4/80-PE (#123110, Biolegend, CA, USA). Stained cells were washed in 1mL PBS containing 2% FBS by centrifugation at 1000 rpm for 5 min, resuspended in buffer, and then detected by flow cytometry. Instrument compensation was performed prior to data acquisition using an antirat/hamster BD CompBeads^TM^ (#552845, Becton, Dickinson and Company) according to the manufacturer's recommendations. Aqua-positive cells were excluded from the analysis. Conventional macrophages were defined as CD11b^+^/F4/80^+^. FlowJo software was used to analyze the data.

### Migration assay

Migration assays were performed using 24-well transwell inserts with 8.0 μm microporous membranes (Corning, Beijing, China). THP-1 cells were preincubated with PMA (100 ng/mL) for 24 h before stimulation with LPS (100 ng/mL) for 6 h. RAW264.7 cells were treated with LPS (100 ng/mL) for 6 h. These two cell lines were starved in FBS-free DMEM for 24 h, and then treated with DPI (10^-13^ or 10^-14^ M) or DMSO for 24 h. Supernatant was collected and added to the lower chamber to serve as a chemoattractant. THP-1 and RAW264.7 cells were resuspended in 100 μL serum-free medium and then seeded into the upper chamber at a density of 4×10^5^/well. Cells were allowed to migrate for 12 h at 37 °C in 5% CO2 and then fixed and stained with crystal violet (#MA0150, Meilunbio). The number of migrated cells in three random fields per well was counted. Images were acquired using a microscope (ZEISS, Jena, Germany) with a charge-coupled device (CCD) camera.

### Western blotting

Total protein was extracted with radioimmunoprecipitation assay (RIPA) buffer containing protease inhibitor cocktails (#C14012, Bio-tool, Beijing, China) and phosphatase inhibitor (#B15001-A&B, Biomake, Shanghai, China). Proteins were separated by SDS-polyacrylamide gel electrophoresis (10%) and transferred to polyvinylidene fluoride (PVDF) membranes (Bio-Rad, CA, USA), as described previously. Membranes were probed with the antibodies listed in Table 1. Signals were visualized using enhanced chemiluminescence (Amersham Imager 600, GE, MA, USA).

### RNA extraction and analysis

Total RNA from cells and tissues was isolated using RNAiso Plus reagent (#9109, Takara, Tokyo, Japan) according to the manufacturer's protocol. The cDNA was synthesized using the All-in-one cDNA Synthesis Super Mix Kit (#B24408, Biomake) for cellular RNA and High Capacity cDNA Reverse Transcription Kit (#4368814, Thermo Fisher Scientific,MA,USA) for tissue RNA. Quantitative real-time polymerase chain reaction (qRT-PCR) was performed using SYBR Green Master Mix (#CW0659, Cwbiotech, Beijing, China). The target gene expression was normalized to that of the actin gene and TATA-box-binding protein (Tbp) gene. Primer sequences are listed in Table 2.

### Statistical analysis

Data are presented as mean ± standard error of mean (SEM). Differences were analyzed using Student's t test or one-way ANOVA. All survival analyses were performed using the log-rank test. All statistical analyses were performed using Prism (GraphPad Software, CA, USA). A *P* value of < 0.05 was considered to determine statistical significance.

## Results

### An ultralow dose of DPI alleviates DSS-induced murine colitis

**Figure 1:**
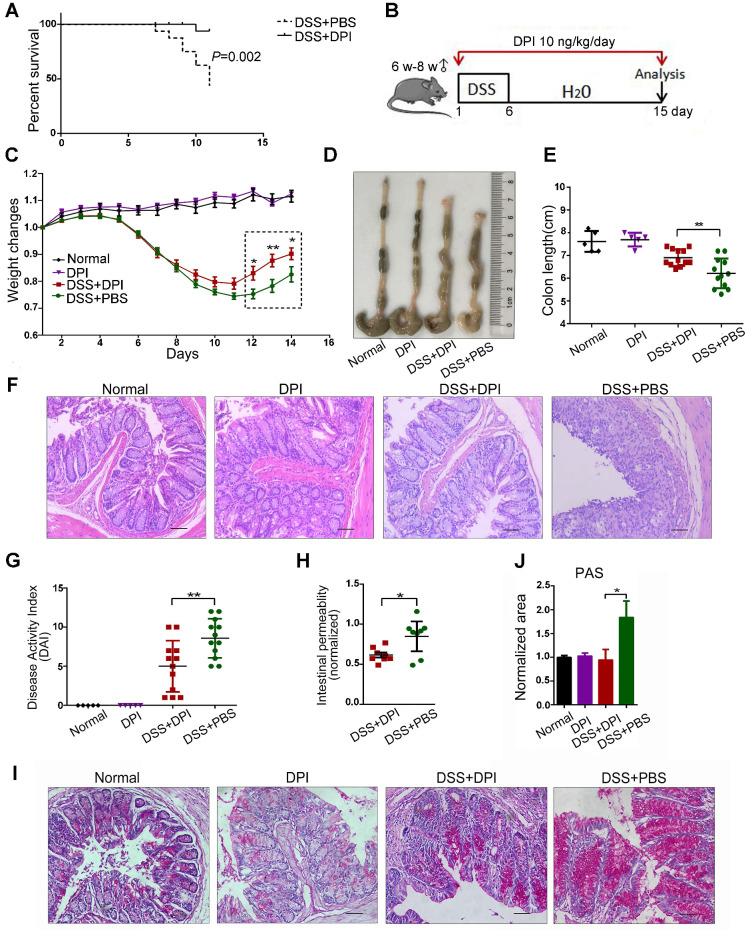
** An ultralow dose of DPI alleviates DSS-induced murine colitis.** (A) DPI improved the survival rate of mice with DSS-induced lethal colitis. The Kaplan-Meier survival curves were verified by the log-rank test. P = 0.002, n = 15 in each group. (B) Schematic overview of DSS-induced acute colitis. (C) Weight changes were recorded throughout the progression of colitis. (D) The colon was photographed, and (E) the colon lengths were measured. (F) Representative H&E staining of mouse colon (bar = 100 µm). (G) The disease activity index was evaluated on day 14. n = 5 for the control groups, n = 12 for the DSS-induced colitis groups. (H) Intestinal gut barrier permeability was assessed by the FITC-dextran assay. The FITC-dextran concentrations detected in the serum of the colitis groups were normalized to those of the normal group. n = 8 in both DSS+PBS and DSS+DPI groups. (I) PAS staining in the colon of each group. (J) The positive area values were normalized to those of the normal group. ∗P < 0.05, ** P < 0.01.

To determine the effective dosage of DPI in a mouse colitis model, we conducted an exploratory dose-response study with daily i.p. injections of DPI at 10 ng to 1 mg/kg. Survival varied noticeably in each group (Figure S1). Mice treated with DSS+PBS and 10 ng/kg DPI showed almost no difference in the survival rate (86.7% vs 90.9%), whereas most mice treated with 1 mg/kg (76.9%) and 1 μg/kg (41.7%) DPI died within 15 days. Notably, in the lethal colitis model (Figure 1A), mice treated with 10 ng/kg DPI showed improvement and prolonged survival compared to those in the control group (93.3% vs 37.5%). Therefore, we selected 10 ng/kg of DPI as the experimental dose for this study.

Next, to evaluate the protective effects of ultralow doses of DPI in the early stages of CAC, mice were treated with a single 6-day DSS cycle, followed by 9 days of administration of normal drinking water; the body weight and presence of diarrhea and fecal blood were assessed every day (Figure 1B). As previously reported, weight loss is an indication of severity in colitis 24. As shown in Figure 1C, both groups of mice had similar weight loss until day 8. After that, the DPI treatment group recovered faster during days 11-15. In addition, DPI treatment significantly limited the DSS-induced colonic shortening (Figure 1D-E). Histologically, the characteristics of colitis were alleviated in the DPI treatment group, except for minor erosions and presence of some infiltrating leukocytes. In contrast, the control mice displayed severe damage to the intestinal epithelium and crypt with extensive erosion and inflammatory cell infiltration in the colonic mucosa (Figure 1F). The disease activity index (DAI) score is a clinical parameter reflecting the rectal bleeding and stool consistency. Treatment with DPI significantly decreased the DSS-induced DAI score from 8.60 ± 0.70 to 5.00 ± 0.98 (Figure 1G).

Intestinal inflammation is often associated with a compromised intestinal epithelial barrier and increased intestinal permeability 25,26. Considering that an ultralow dose of DPI could inhibit intestinal inflammation, we next investigated the integrity of the intestinal epithelial barrier using a FITC-dextran assay. Significantly higher plasma FITC-dextran levels were detected in control mice than in the DPI-treated mice, suggesting that high intestinal permeability may be linked to alterations in host defense mechanism (Figure 1H). Goblet cells are columnar epithelial cells characterized by the secretion of mucins, and they function as an intestinal barrier that protects the epithelium 27. PAS staining revealed decreased level of mucins in the colon of DPI-treated mice (0.94 ± 0.22 vs 1.84 ± 0.34) (Figure 1I-J).

Additionally, the activation of Wnt/ β-catenin signaling plays a central role in the occurrence and development of colorectal cancer 28. Therefore, we examined the expression of target genes regulated by β-catenin in the colitis-affected tissues. The results of qPCR results showed that the mRNA levels of c-myc, cyclin D1 (CCND1), tenascin C (TNC), Axin2, and β-catenin in the DPI treatment group were significantly down- regulated (Figure S2).

Collectively, these results suggest that an ultralow dose of DPI can ameliorate DSS-induced colitis.

### Ultralow dose DPI suppresses inflammatory responses and ROS production

**Figure 2:**
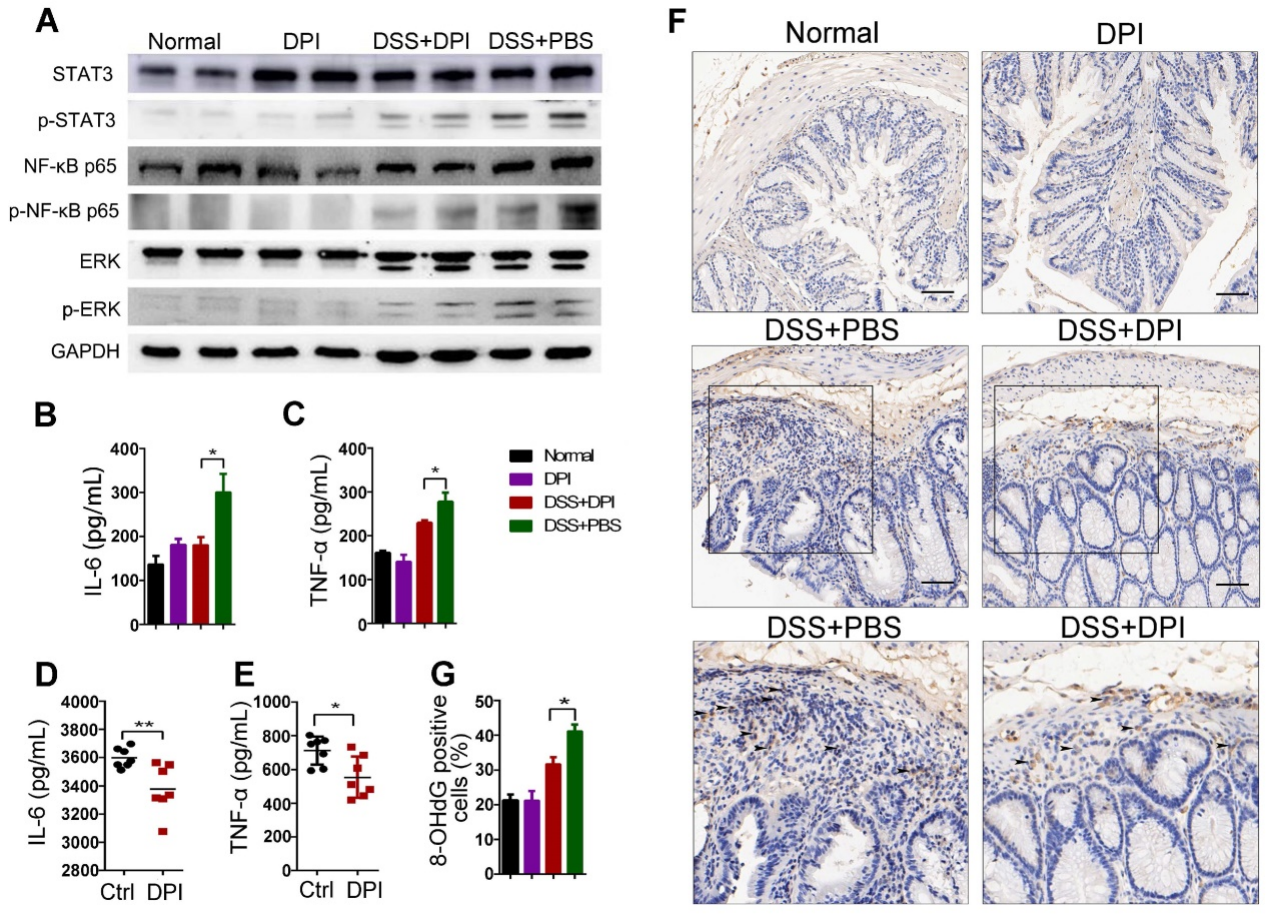
** Ultralow dose DPI suppresses inflammatory responses and ROS production.** (A) Analysis of the indicated proteins in the colonic lamina propria of the DSS-induced colitis model. The colonic lysates were collected on day 15 after DSS challenge. Serum levels of IL-6 (B) and TNF-α (C) in the DSS-induced colitis model (n = 5 per group). Four hours after LPS injection, the serum levels of IL-6 (D) and TNF-α (E) were determined by ELISA (n = 7 per group). (F) Representative images of 8-OHdG staining in the colon of each group are shown (black arrow indicates the positive area (brown)). Bar = 100 µm. (G) The percentage of 8-OHdG positive cells/ visual field in the colonic tissues was enumerated using Image-Pro Plus 5.0. Three fields per mouse were scored. *P < 0.05, **P < 0.01.

As described above, an ultralow dose of DPI exhibited a protective effect against colitis. It is well known that multiple signaling pathways involving STAT3, MAPK, and NF-κB are activated in the pathogenesis of IBD and CRC 29-31. We then analyzed whether DPI could regulate the crucial inflammatory-related pathway in the lamina propria cells of the colitis model by western blotting. The results demonstrated that the activation of extracellular signal-regulated kinase 1/2 (ERK1/2), STAT3, and NF-κB p65 increased in the control mice after DSS treatment. In contrast, DPI treatment decreased the phosphorylation of ERK1/2, STAT3, and NF-κB p65 (Figure 2A). To further explore the effect of ultralow-dose DPI on cytokine production in mice with DSS-induced colitis, the protein levels of cytokines in the serum of treated mice were measured by ELISA. As shown in Figure 2B-C, DSS treatment significantly induced the production of IL-6 and TNF-α, while DPI treatment markedly reduced the levels of these cytokines. We also challenged mice with LPS, which caused systemic inflammation, and then collected blood samples to measure plasma IL-6 and TNF-α production. Similar to the results in the colitis model, the ultralow dose of DPI significantly decreased the plasma levels of IL-6 and TNF-α compared with those in the control group (Figure 2D-E).

ROS are involved in signal transduction, interact directly with genomic DNA, and damage tumor-related genes that control cell growth and differentiation 32,33. A previous study demonstrated that DPI at subpicomolar concentrations (10^-13^ to 10^-14^ M) was capable of inhibiting superoxide production in primary midbrain neuron-glia cultures. To investigate whether DPI inhibited ROS generation in an acute colitis model at a subpicomolar concentration, we measured ROS levels in vitro and in vivo. RAW264.7 and THP-1 cell lines were labeled with DCFH-DA and then analyzed by flow cytometry. As shown in Figure S3, DPI treatment significantly reduced the increase in LPS-induced ROS fluorescence intensity. In addition, we determined the expression of hydroxy-2′-deoxyguanosine (8-OHdG), a well-known marker of oxidative damage, in colon tissue 34. As shown in Figure 2F-G, the 8-OHdG-positive cells in the DPI treatment group were significantly lower than those in the control group (31.6% vs 41.2%), suggesting that an ultralow dose of DPI inhibited DSS-induced ROS generation.

### An ultralow dose of DPI inhibits the infiltration and migration of macrophages

**Figure 3:**
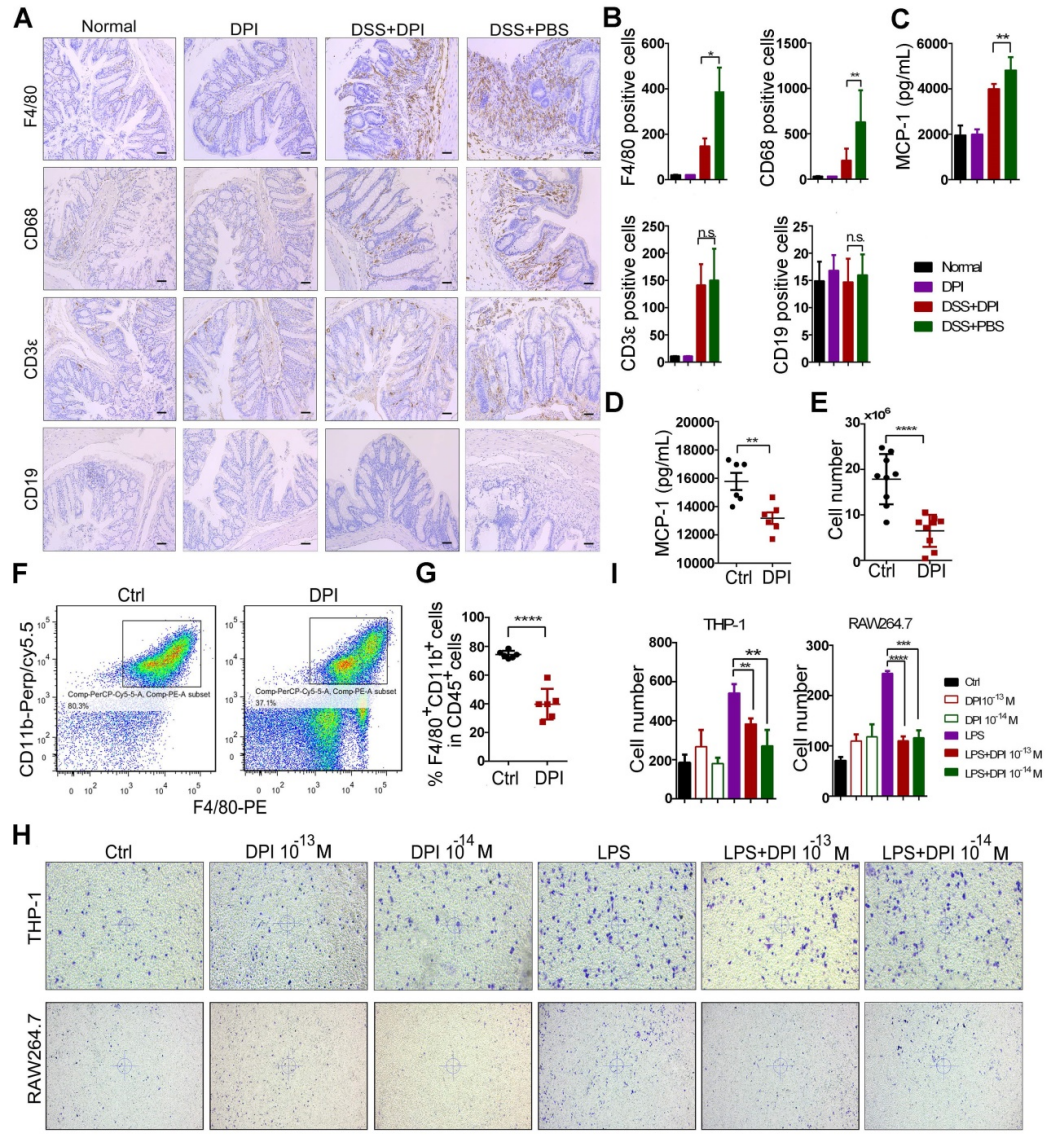
** An ultralow dose of DPI inhibits the infiltration and migration of macrophages.** Immunohistochemical staining of F4/80, CD68, CD19, and CD3ε in the paraffin-embedded sections (Bar = 100 µm) and (B) quantification of the positive cells by Image-Pro Plus 5.0. (C) Serum levels of MCP-1 were measured by ELISA on day 15 after the DSS treatment (n = 5 per group). (D) MCP-1 levels in serum of mice in the peritonitis models (n = 6 per group). (E) The number of peritoneal macrophages isolated from the control and DPI groups in the thioglycolate-elicited peritonitis models (n = 9 per group). (F) The expression of the phenotypic markers of macrophages was determined by flow cytometry. Scatter gram of cells expressing F4/80 and CD11b in the control and DPI groups. Peritoneal cells were collected on day 4 after the thioglycolate injection. (G) The proportion of macrophages in CD45^+^ cells (n = 6 per group). (H) Images showing the migration of the THP-1 and RAW264.7 cells. Cells were incubated with supernatant containing DPI for 12 h at 37 °C. The experiment was repeated three times. (I) The cell number in each group in the migration assay. The results are expressed as mean ± SEM. *P < 0.05, **P < 0.01, ***P < 0.001, ****P < 0.0001.

The intestinal accumulation of immune cells contributes to the initiation of inflammation-associated colon cancer 35. Thus, we analyzed the effect of DPI treatment on colonic immune cell infiltration. The expression of macrophage markers (F4/80, CD68) and other immune cell markers, such as the B cells (CD19) and T cells (CD3ε), was evaluated by immunostaining of the colon sections. After DSS treatment, the number of T cells and macrophages in the intestinal mucosa increased significantly. Compared with that in the control group, the number of macrophages in the DPI-treated group was reduced by up to 1.5 fold, whereas the number of B cells was unchanged (Figure 3A-B).

It is well known that macrophage recruitment to the site of inflammation depends on MCP-1^36^. Therefore, we investigated whether DPI could regulate the expression of MCP-1 and affect the migration of macrophages. As shown in Figure 3C, the serum MCP-1 levels obviously decreased in the DPI-treated mice. To further confirm the effect of DPI on macrophage migration, we used the thioglycolate-induced mouse peritonitis model 37. As shown in Figure 3D, the plasma MCP-1 levels were also significantly inhibited by DPI. Then, we examined the effects of DPI on macrophage migration. Peritoneal macrophages (PMs) were isolated and counted 3 days after thioglycolate treatment. The results clearly showed that the number of PMs in the DPI-treated group was much lower than that in the control group (6.52×10^6^ vs 17.90 ×10^6^; Figure 3E). Compared with that in the control group, the percentage of macrophages (F4/80^+^ / CD11b^+^) within the CD45^+^ cell population in the DPI group decreased significantly (Figure 3F-G). To examine whether MCP-1 antibody could block the migration of macrophages, we performed a transwell assay in the absence or presence of MCP-1 antibody. The results clearly demonstrated that MCP-1 blockade significantly inhibited the migration of RAW264.7 cells and BMDMs (Figure S4).

To determine the effect of DPI on macrophage migration in vitro, we treated both the THP-1 and RAW264.7 cells with DPI. The results showed that DPI significantly inhibited the LPS-induced migration of RAW264.7 and THP-1 cells (Figure 3H-I), suggesting that an ultralow dose of DPI can regulate the inflammatory tumor microenvironment in DSS-induced colitis.

### An ultralow dose of DPI inhibits the classical activation of macrophages

**Figure 4:**
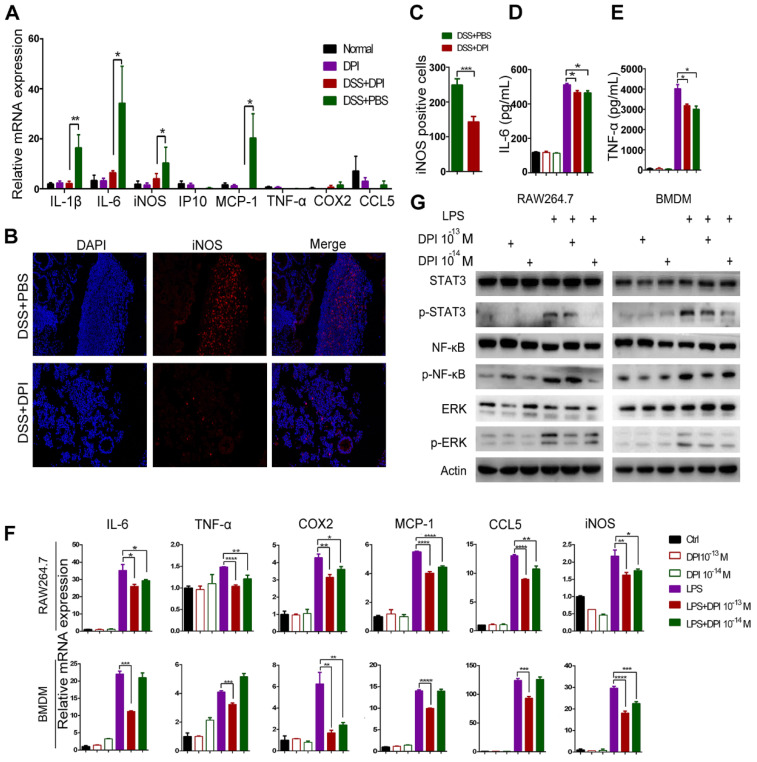
** An ultralow dose of DPI inhibits the classical activation of macrophages.** (A) Analysis of the M1-associated mRNA expression in the colonic lamina propria of DSS-induced colitis mice. The colon samples were analyzed on day 15 after DSS treatment (n = 5 per group). (B) The M1-associated marker, iNOS, was analyzed by immunofluorescence and (C) the number of iNOS^+^ cells/ visual field in the colonic tissues was enumerated. (D) IL-6 and (E) TNF-α levels in the supernatants of BMDMs treated with LPS and DPI for 6 h. M1-associated mRNA levels (F) and protein levels (G) in RAW264.7 cells and BMDMs. Cells were treated with LPS (500 ng/mL) and DPI (10^-13^ or 10^-14^ M) for 6 h and then analyzed by qRT-PCR and immunoblotting with the indicated antibodies. The experiment was repeated three times. The results are presented as mean ± SEM. *P < 0.05, **P < 0.01, ***P < 0.001, ****P < 0.0001.

Macrophages are functionally important in the initiation and promotion of tumorigenesis 23. We have previously shown in an acute colitis model that DPI treatment decreased the colonic macrophage infiltration, which was consistent with a reduction in inflammation. Thus, we hypothesized that DPI treatment could lead to altered macrophage activation in the tumor microenvironment. M1 macrophages are considered to exert pro-inflammatory effects. To evaluate whether an ultralow dose of DPI could inhibit M1 macrophages, we examined the mRNA levels of classical activation-related genes in lamina propria cells. The results showed that DPI remarkably reduced the DSS-induced upregulation of the inflammatory cytokines, including IL-1β, IL-6, iNOS, and MCP-1 (Figure 4A). In contrast, there was no significant difference in the transcriptional levels of CD8^+^ and Treg cell surface markers between DPI-treated and control groups 26 (Figure S5A-B). In addition, the expression levels of the M2 markers, Arg1, CD206, and YM1 in BMDMs of the two groups were similar (Figure S5C-D). We next analyzed the expression of iNOS (M1 marker) and Arg1 (M2 marker) in the colonic mucosa by immunofluorescence staining. The number of iNOS^+^ cells was obviously decreased in the DPI-treated mice (Figure 4B-C). However, there was no significant difference in the number of Arg1^+^ cells between DPI and PBS groups (Figure S5 E-F).

To assess whether DPI resulted in an alteration of the baseline macrophage activation pattern, we isolated BMDMs from mice and stimulated them ex vivo with classical M1 stimuli (LPS) in the presence or absence of DPI. The markers of M1 macrophage activation were then assessed. As expected, ELISA data showed that the secretion of IL-6 and TNF-α in the DPI-treated cell supernatants was significantly lower than that in the control group supernatants (Figure 4D-E). Moreover, the mRNA levels of M1 markers, including IL-6, TNF-α, cytochrome c oxidase subunit II (COX2), MCP-1, C-C motif chemokine ligand 5 (CCL5), and iNOS were decreased in BMDMs isolated from the DPI-treated mice (Figure 4F). The STAT3, NF-κB, and ERK signaling pathways have been implicated in M1 polarization of macrophages. As expected, we found that an ultralow dose of DPI significantly inhibited these pro-inflammatory pathways (Figure 4G).

Taken together, these results clearly show that an ultralow dose of DPI can effectively inhibit the classical activation of macrophages in vitro and in vivo.

### An ultralow dose of DPI prevents the formation and development of CAC

**Figure 5:**
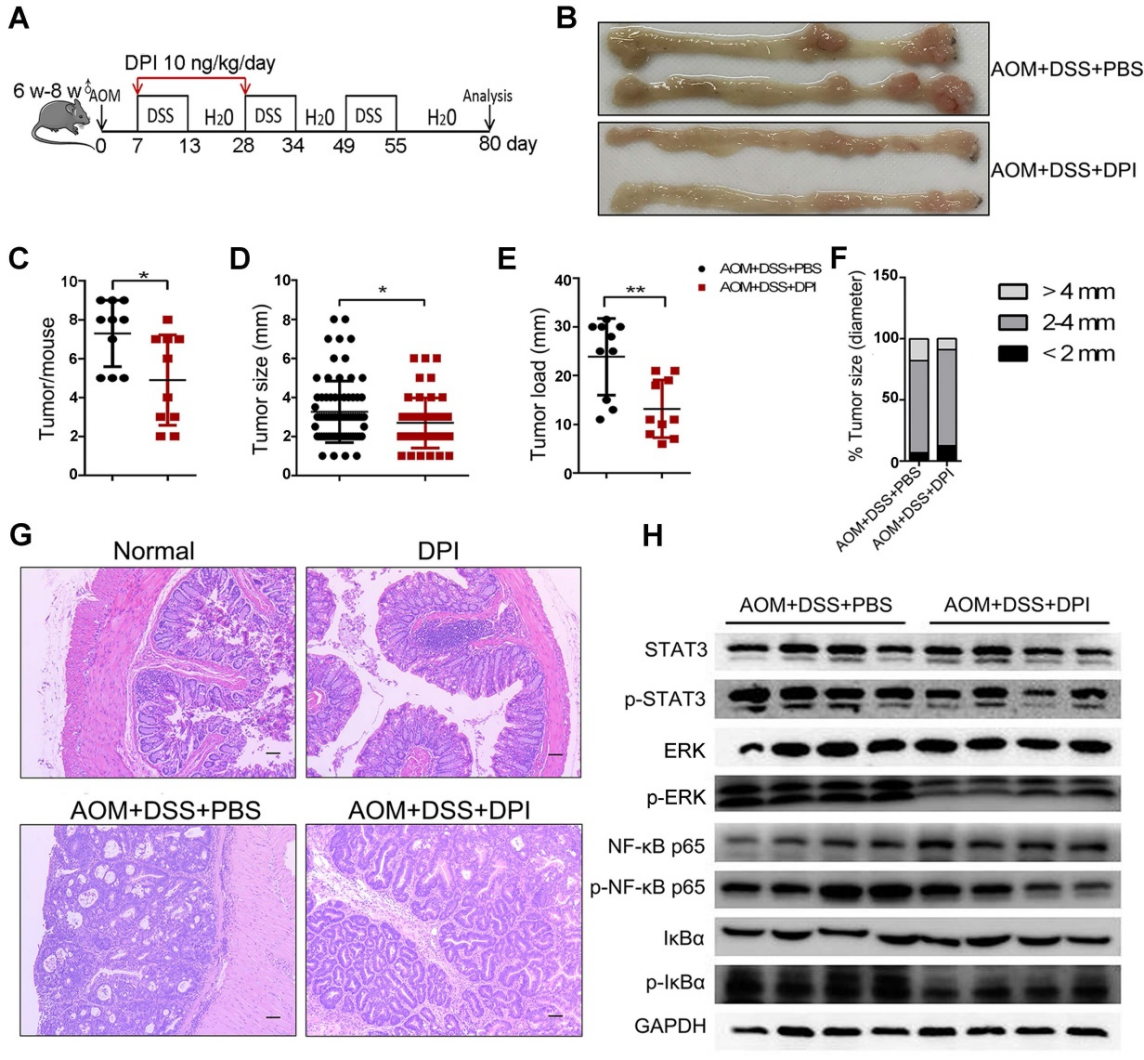
** An ultralow dose of DPI prevents the formation and development of CAC.** (A) Mode pattern of the AOM/ DSS-induced CAC model. (B)Representative photographs of murine colons. (C) Number of tumors per mouse. (D) Tumor size, (E) tumor load, and (F) tumor distribution were measured for each group. (G) H&E staining of tumor morphology (Bar = 100 µm). n = 10 per group. (H) Analysis of the indicated proteins in the colonic intestinal epithelial cells. The experiment was repeated three times. Differences were calculated using a two-tailed Student's t-test. *P < 0.05, **P < 0.01.

To investigate the role of ultralow dose of DPI in CAC tumorigenesis, mice were treated with AOM/ DSS (Figure 5A). Eighty days after AOM injection, mice were euthanized, their colons were resected, and the number of tumors and tumor size were measured. Both the control and DPI-treated mice developed tumors, which were observed primarily in the distal to middle colon (Figure 5B). The DPI-treated mice showed an obvious decrease in the number of tumors as compared to that in the control mice (4.90 ± 2.33 vs 8.50 ± 0.55), and these tumors were smaller than those in the control group mice (Figure 5C-D). Moreover, the average tumor load, defined as the total diameter of all tumors in a given mouse, was notably reduced by the DPI treatment (13.20 ± 5.92 cm vs 23.85 ± 0.83 cm) (Figure 5E). The tumor size distribution was also different between the DPI-treated and the control mice, for example, the number of large size tumors (> 4 mm) was significantly decreased in the DPI group compared to that in the control mice (9.0% vs 17.8%), whereas the number of small tumors (< 2 mm) was increased (12.5%vs 6.9%) (Figure 5F). Histologically, most tumors in the DPI-treated mice were low-grade intraepithelial neoplasia (IEN) with glandular structures. However, more aggressive adenocarcinomas were found in the control group (Figure 5G). Intriguingly, when DPI was administered only in the late stage in the CAC model (the last cycle of DSS water treatment, from days 49-70), no antitumor effects of DPI were observed (Figure S6). These findings indicate that an ultralow dose of DPI inhibits the inflammation-associated colon tumorigenesis in the early developmental stages.

To further understand the possible mechanism by which ultralow dose DPI reduces colon cancer, we evaluated the activation of tumor-promoting signaling pathways, such as STAT3, MAPK, and NF-κB, in IECs. Western blotting data showed that the phosphorylation of NF-κF p65, IκBα, STAT3, and ERK in the DPI-treated group was much lower than those in the control group, indicating a significant inhibitory effect of DPI on the signaling pathways involved in promoting malignant transformation of IECs (Figure 5H).

Since DPI can inhibit the occurrence of CAC induced by AOM/ DSS, we evaluated whether DPI has a direct inhibitory effect on the growth of CRC cells. As shown in Figure S7A-B, an ultralow dose of DPI had no effect on the size of xenograft tumors in mice. Similarly, DPI did not change the proportion of Ki67 positive cells (Figure S7C-D). According to the research recommendations, we used subpicomolar concentrations of DPI in the cell experiments 19. As shown in Figure S7E-J, DPI at the recommended dose (10^-5^ M) significantly inhibited cell proliferation and promoted apoptosis. However, DPI at 10^-13^ M and 10^-14^ M had no evident effect on proliferation or apoptosis of the CRC cells. It should be noted that treatment with 10 ng/kg DPI for 3 weeks did not produce any toxicity based on the H&E staining of various organs (Figure S8).

Taken together, the above results indicate that ultralow dose DPI can inhibit the progression of CAC by inhibiting acute colitis, whereas it has no direct killing effects on CRC cells.

## Discussion

Inflammation is currently considered a hallmark of cancer and is associated with different stages of tumorigenesis, including initiation, progression, and metastasis 38. A previous study revealed that treatment with DPI had a protective effect on the pro-inflammatory activity of bacterial endotoxins in mouse colonic epithelial cells during acute and chronic colitis 39. Moreover, DPI enhanced the phagocytosis of E. coli by murine primary macrophages, which depended on the intracellular calcium levels. Activation of p38 MAPK facilitated bacterial elimination and amelioration of inflammation leading to improved survival of E. coli-infected mice 40. In addition, DPI significantly decreased the growth of both HT-29 and LS-174T human tumor xenografts 41. However, the role of DPI in regulating the initiation and progression of CAC in more typical physiological settings remains unclear.

Here, we explored the role of DPI in murine colitis and CAC. The results show that DPI can effectively alleviate DSS-induced colitis (Figure 1) by inhibiting inflammatory responses, such as macrophage recruitment (Figure 3). DPI also decreased the production of several protumoral cytokines (IL-6 and TNF-α) and chemokines (MCP-1) (Figure 2-3). It is worth noting that the DPI dose used in this study (10 ng/kg) was extremely low. Several studies have demonstrated that blocking IL-1β or TNF-α or using IL-17-deficient mice markedly attenuates the colonic inflammation and development of colorectal cancer 42-44, indicating that these cytokines are the key factors responsible for the local inflammation. Our study clearly shows that the expression of protumoral cytokines in the plasma and colonic lamina propria was dramatically inhibited by DPI (Figure 2, 4). During the development of CAC, the presence of a proinflammatory intestinal microenvironment favors the activation of oncogenes 45. STAT3, NF-κB, and MAPK are crucial inflammation and cancer-associated pathways. The NF-κB signaling in macrophages maintains the innate immune response and induces an over-activated immune response in inflammatory diseases 46. Reduced tumor growth upon ablation of STAT3 and blockade of IL-6/IL-6 signaling has been previously reported in CAC 47. Gupta et al. also demonstrated that the pharmacological inhibition of the MAPK pathway reduced the growth of colon tumors 48. Deletion of IKKβ in myeloid cells was associated with a less marked reduction in the adenoma incidence but was associated with reduced adenoma size and reduced expression of genes encoding proinflammatory cytokines, such as IL-1β, IL-6, and TNF-α 49. Our study found that DPI significantly inhibited STAT3, NF-κB, and MAPK pathways in the colonic lamina propria indicating a DPI-mediated suppression of tumorigenic inflammation in mouse colon (Figure 2). In this study, our data demonstrated that DPI inhibits CAC tumorigenesis through the suppression of inflammation in the lamina propria.

Macrophages are crucial cells present in the microenvironment that maintain the gastrointestinal homeostasis, and are also important to prevent the invasion of many pathogens 50. In IBD patients and mouse models of colitis, M1 macrophages infiltrate the lamina propria, shifting the balance in the macrophage pool towards a proinflammatory population and compromising the epithelial integrity via the secretion of cytokines and effector molecules, such as ROS or nitric oxide, ultimately leading to aggravation of the intestinal inflammation 51,52. Considering that the macrophage polarization plays a key role in colitis, it is necessary to know whether DPI can ameliorate the inflammatory response by influencing macrophage polarization. In this study, we discovered that DPI inhibited M1 polarization in the lamina propria of inflamed colonic tissue (Figure 4A-C). Moreover, DPI inhibited the expression of M1-associated genes and cytokines in the LPS-stimulated RAW264.7 and BMDM cells (Figure 4D-E).

Inflammatory cells, especially macrophages, are a major source of ROS generation, which can induce DNA damage and genomic instability. In inflammatory diseases, such as UC, vast amounts of ROS produced by macrophages and other inflammatory cells cause a direct damage to the epithelial cells 53. Antioxidants, such as N-acetylcysteine, can protect against CAC in mouse models 54. Our results demonstrated that DPI can reduce the number of 8-OHdG-positive cells, suggesting a decrease in ROS production in the colon (Figure 2F), which is in accordance with the in vitro data (Figure S3). A previous study demonstrated that DPI at subpicomolar concentrations (10^-13^ to 10^-14^ M) exhibits specific effects against NOX2 in primary midbrain neuron-glia cultures 55. Bao et al. reported that gp91^phox^ (a subunit of the NOX2 complex)-deficient mice were less susceptible to DSS-induced acute colitis; this may be due to a decrease in the oxidative burst in the intestine, thereby reducing tissue damage 56. Huang et al. demonstrated that mice with NOX2 subunit knockout exhibited reduced microglial activation, attenuated production of the proconvulsive cytokines, IL-1β, TNFα, and IL-6, and lower seizure susceptibility to pentylenetetrazole following LPS-induced inflammation as compared to that of the wild-type mice. Similarly, post-treatment with DPI following LPS also attenuated the activation of microglia and expression of these proconvulsive cytokines in the mouse brain 57. In this study, we found that ultralow dose DPI can ameliorate DSS-induced colitis, which may be due to the reduction in macrophage infiltration, classical polarization, and ROS generation. This phenotype was similar to that observed in the NOX2 deficient mice. However, there is also a contrary report showing that the DPI-induced enhancement of phagocytosis was not related to NOX2 or ROS, but depended on the increase of intracellular calcium and activation of the p38 MAPK signaling pathway 58. Therefore, we cannot rule out if DPI can inhibit the inflammatory response by regulating the NOX2 independent mechanism, which needs further study.

Although there are relevant methods for the early detection of CRC 59,60, the precise pathogenesis of IBD-related colorectal tumorigenesis remains unclear. However, the continuous activation of crosstalk between the lamina propria cells and intestinal epithelial cells is responsible for the malignant transformation 61,62. Our study clearly demonstrates that ultralow doses of DPI can prevent AOM/ DSS-induced adenomatous polyps (Figure 5). It seems that DPI inhibited the tumor initiation after the inflammatory event but did not inhibit the progression of CRC. As shown in Figure S6, we found that there was no difference in the number of tumors and tumor size between the treatment and the control groups when DPI was administered after the formation of CAC. Therefore, using only ultralow dose DPI in the early stages of CAC can reduce macrophage infiltration and M1 polarization, thus reducing colitis and the occurrence of tumors. In addition, it is well known that tumor-associated macrophages (TAMs) can promote tumor cell invasion and metastasis, thus promoting tumor angiogenesis 63. Whether DPI could reduce tumor metastasis by inhibiting the migration of TAMs remains to be further investigated. Additionally, it is important that there is no evidence of toxicity in mice with long-term use of ultralow doses of DPI (Figure S8). Therefore, the ultralow doses of DPI have high efficiency and low toxicity and may become a potential treatment method in clinic in the future.

**Figure 6:**
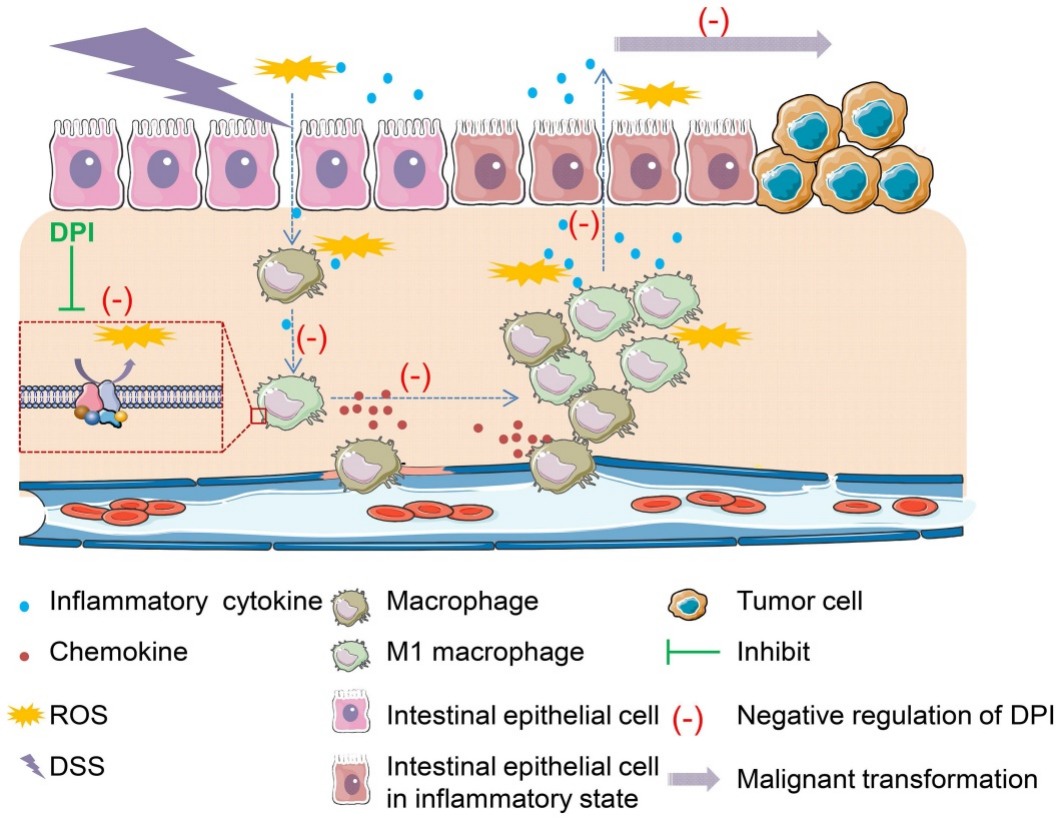
** Proposed model showing how an ultralow dose of DPI attenuates the progression of colitis-associated colorectal cancer.** ROS is a major stimulant that activates macrophages into pro-inflammatory macrophages. The self-propelling malignant cycle between the damaged epithelial cells and activated macrophages is critical in driving the progression of malignant transformation into colitis-associated colorectal cancer. As an inhibitor of NADPH oxidase, DPI has no toxicity when used at ultralow doses. It can reduce ROS levels and inhibit the downstream pathways involved in chronic inflammation. Once the damaged macrophages or epithelial cells or both are inhibited, the progression of the self-propelling malignant cycle is halted.

In conclusion, this study provides convincing evidence that an ultralow dose of DPI can effectively reduce the macrophage-mediated inflammatory response, and thus inhibit the occurrence and development of CAC (Figure 6). Our findings may provide a novel and effective therapeutic agent for the treatment of colitis and CAC in future.

## Supplementary Material

Supplementary figures and tables.

## Figures and Tables

**Table 1:** Antibodies used in this study

Antibody	Supplier	Cat.No.	Dilution	Application
STAT3	Cell signaling technology	9139	1:1000	WB^a^
p-STAT3	Cell signaling technology	9145	1:1000	WB
NF-κB p65	Cell signaling technology	8242	1:1000	WB
p- NF-κB p65	Cell signaling technology	3033	1:1000	WB
IκBα	Cell signaling technology	4814	1:1000	WB
p- IκBα	Cell signaling technology	2859	1:1000	WB
Erk	Cell signaling technology	4695	1:1000	WB
p-Erk	Cell signaling technology	4370	1:1000	WB
Arg1	Cell signaling technology	93668	1:1000	WB
Ym1	Abcam	ab192029	1:1000	WB
CD206	Abcam	ab46493	1:1000	WB
Actin	Cell signaling technology	8457	1:1000	WB
GAPDH	Elabscience	EAB20032	1:1000	WB
Ki67	Abcam	ab16667	1:200	IHC^b^
F4/80	Cell Signaling Technology	70076	1:400	IHC
CD19	Cell Signaling Technology	90176	1:200	IHC
CD68	Abcam	ab125212	1:800	IHC
CD3ε	Biolegend	362701	1:200	IHC
anti-8-OHdG^c^	Abcam	ab48508	1:50	IHC
iNOS	Novus	NB300-605	1:50	IF^d^
Arg1	Cell Signaling Technology	CST93668	1:200	IF

^a^WB: Western blotting. ^b^IHC: Immunohistochemistry. ^c^8-OHdG: 8-hydroxy-2′-deoxyguanosine. ^d^IF: immunofluorescence.

**Table 2:** Primer sequences of RT-PCR

Genes	Sequences
Axin2	F: TAGGTTCCGGCTATGTCTTTG
	R: TGTTTCTTACTCCCCATGCG
c-myc	F:TAGTGCTGCATGAGG AGACA
	R: GGT TTGCCTCTTCTCCACAG
CD44	F:GTC TGCATCGCGGTCAAT AG
	R:GGT CTC TGATGG TTCCTTGTTC
CyclinD1	F: GCACAACGCACTTTCTTTCCA
	R: CGCAGG CTTGACTCCAGAAG
COX2	F: CAGCCAGGCAGCAAATCCT
	R: CTTATACTGGTCAAATCCTGTGCTCA
CCL5	F: GTGCTCCAATCTTGCAGTCG
	R: AGAGCAAGCAATGACAGGGA
CD2	F:TGTATGGCACAAATGGGATG
	R:CAGTGGATCATGGGCTTT
CD8α	F:TGCTGTCCTTGATCATCACTCT
	R:ACTAGCGGCCTGGGACAT
CD8β	F:GCACTGAGGGGAACAGTGTC
	R:GACGAAGGGGTCTGAATGAG
CTLA4	F:TCACTGCTGTTTCTTTGAGCA
	R:GGCTGAAATTGCTTTTCACAT
FOXP3	F:ACCACACTTCATGCATCAGC
	R:ATCATGGCTGGGTTGTCC
GZMα	F:CTTTTTCTTCTGCTTATTCCTGAAG
	R:CGGTCTTGAGTGAGGAACAAC
GZMβ	F:GCTGCTCACTGTGAAGGAAGT
	R:TGGGGAATGCATTTTACCAT
GZMκ	F:TCATGGGCTCTGGTTTCC
	R:TCCCTCCAATAATTTCAGTATGG
iNOS	F: CAGCTGGGCTGTACAAACCTT
	R: CATTGGAAGTGAAGCGTTTCG
IP10	F: CCAAGTGCTGCGTCATTTTC
	R: GGCTCGCAGGGATGATTTCAA
IL-1β	F: GCTGAAAGCTCTCCACCTCA
	R: AGGCCACAGGTATTTTGTCG
IL-15	F:CAGAGGCCAACTGGATAGATG
	R:ACTGTCAGTGTATAAAGTGGTGTCAAT
IL-6	F: GAGGATACCACTCCCAACAGACC
	R: AAGTGCATCATCGTTGTTCATACA
MCP-1	F:CGGAACCAAATGAGATCAGAA
	R: TGTGGAAAAGGTAGTGGATGC
Tbp	F:ACCGTGAATCTTGGCTGTAAAC
	R:GCAGCAAATCGCTTGGGATTA
TNC	F: ACCATGCTGAGATAGATGTTCCAAA
	R: CTTGACAGCAGAAACACCAATCC
TNF-α	F: TACTGAACTTCGGGGTGATCGGTCC
	R: CAGCCTTGTCCCTTGAAGAGAACC
TGF-β1	F:CACAGTACAGCAAGGTCCTTGC
	R:AGTAGACGATGGGCAGTGGCT
ZAP70	F:TCATGCTGGTCATGGAGATG
	R:GCTCACAGGCATCTCCTCCT
β-Actin	F: AGAGGGAAATCGTGCGTGAC
	R: CAATAGTGATGACCTGGCCGT
β-catenin	F: GCTGACCTGATGGAGTTGGA
	R: GCTACTTGCTCTTGCGTGAA

## Abbreviations

PBS: phosphate-buffered saline
MPTP: 1-methyl-4-phenyl-1,2,3,6-tetrahydropyridine
MK: MAPK-activated protein kinase
2Chil3: chitinase-like 3
MR: mannose receptor
iNOS: nitric oxide synthase
IFN: interferons
CRC: colorectal cancer
CD: Crohn's disease
TNF: tumor necrosis factor
IL: interleukins
PCR: polymerase chain reaction
NADPH: nicotinamide adenine dinucleotide phosphate
DPI: diphenyleneiodonium
CAC: colitis-associated cancer
DSS: dextran sodium sulfate
AOM: azoxymethane
ROS: reactive oxygen species
STAT3: signal transducer and activator of transcription 3
MAPK: mitogen-activated protein kinases
NF-κB: nuclear factor kappa B
IBD: inflammatory bowel disease
UC: ulcerative colitis
LPS: lipopolysaccharide
NOX2: NADPH oxidase 2
MCP-1: monocyte chemoattractant protein 1
