# Supplementary material for: An ultralow dose of the NADPH oxidase inhibitor diphenyleneiodonium (DPI) is an economical and effective therapeutic agent for the treatment of colitis-associated colorectal cancer
Source: Theranostics. 2020 May 20;10(15):6743–57. doi: 10.7150/thno.43938 (PMC7295061; doi:10.7150/thno.43938)
Supplement: Supplementary file 1 — Supplementary figures and tables. [file thnov10p6743s1.pdf]

Supplementary material

Measurement of Cellular ROS

Intracellular ROS were detected using a cell permeable ROS-sensitive dye, chloromethyl-20, 70-dichlorofluorescein diacetate (DCFH-DA, Sigma-Aldrich). According to the manufacturer's instructions, RAW264.7 and THP-1 cells (1×10^6 cells/well in 6-well plates) were simultaneously stimulated with LPS (500 ng/mL, #L4391,Sigma) and DPI (10^{-13} M, 10^{-14} M) for 12 h. Then, the cells were rinsed with PBS and incubated with 10 μM DCFH-DA for 30 min at 37 ℃. Next, the cells were washed with PBS and incubated with Live/Dead Aqua (#L34957,Invitrogen) for 20 min at room temperature. After washing twice with PBS, the cells were detected by a BD FACSCalibur™ flow cytometry (Becton, Dickinson and Company). Aqua-positive cells were excluded from analysis, the data were analyzed by FlowJo Software.

MCP-1 antibody inhibiting cell migration assay

To measure MCP-1 antibody inhibiting migration, RAW264.7 or BMDM were treated with LPS (100 ng/mL) for 6 h, and then cells were cultured in FBS-free DMEM with 0.5 μg/mL or 5 μg/mL MCP-1 antibody (#ab25124, Abcam) in lower chamber. RAW 264.7 and BMDM in FBS free DMEM (0.1 mL, 2×10^5 cells/mL) were added in upper chamber for incubation 15 h

at 37 ℃. The number of migrated cells in 3 random fields per well were counted by software (Image-Pro Plus 5.0). Images were taken using a microscope (ZEISS) with a CCD camera.

Subcutaneous tumor model

6- to 8-week-old C57BL/6 mice were injected subcutaneously with 1×10^6 MC38 cells. Three days later, mice were treated with intraperitoneal injection of PBS or DPI (10 ng/kg) once daily until the mice were euthanized on the 17th day. Tumor size was measured every day and tumor volume was calculated by 0.523 × (length × width× width).

Apoptosis assay

An annexin V-fluorescein isothiocyanate (FITC)/propidium iodide (PI) cell apoptosis kit was used according to the instructions (Multi sciences, #AP101, China). Briefly, cells (4×10^5 cells/well in 12-well plates) were treated with DPI (10^{-5} M, 10^{-13} M, 10^{-14} M) for 24 h, washed twice with PBS, and resuspended in the binding buffer provided in the kit. After this, 5 μL Annexin V-FITC and 10 μL PI were mixed with the cells. Following 5 min of incubation at room temperature in the dark, mixtures were analyzed using a BD FACSCalibur™ flow cytometry (Becton, Dickinson and Company).

Proliferation assay

CCK-8 assay was performed to detect the effect of DPI on cell proliferation according to manufacturer's instructions. In brief, 4×10^3 cells with 100 μL

DMEM medium containing DMSO or DPI (10^{-5} M, 10^{-13} M, 10^{-14} M) were seeded in 96-well plates in triplicates, 10 μL CCK-8 solution was added to each well and incubated for 2 h at 37 ℃ with 5% CO_2. The absorbance of optical density 450 nm was measured using Synergy H4 Hybrid Reader (Biotek).

Supplementary Figure 1

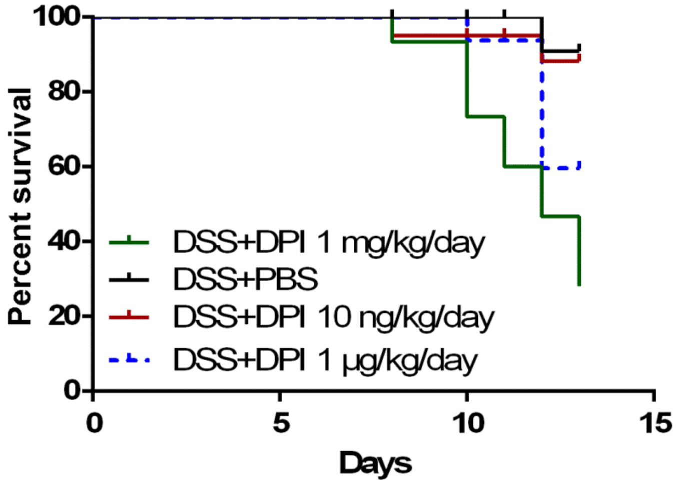

Supplementary Figure 1. The survival rate of mice with DSS-induced colitis treated with different doses of DPI

Supplementary Figure 2

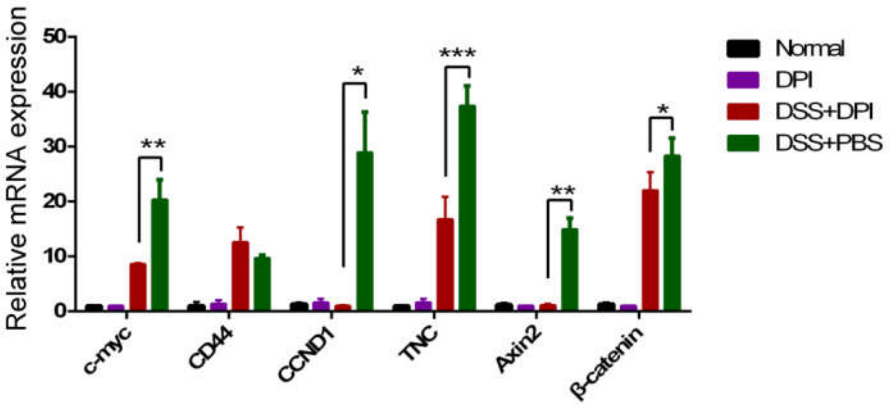

Supplementary Figure 2. Ultralow dose DPI can protect epithelial cells. Relative mRNA expression of β-catenin target genes in the colon tissues was determined by qRT-PCR and the gene expression was normalized to the levels of TATA-box-binding protein (Tbp). The colon samples were analyzed on day 15 after DSS treatment (n = 7 per group). *$P < 0.05$, **$P < 0.01$, ***$P < 0.001$.

Supplementary Figure 3

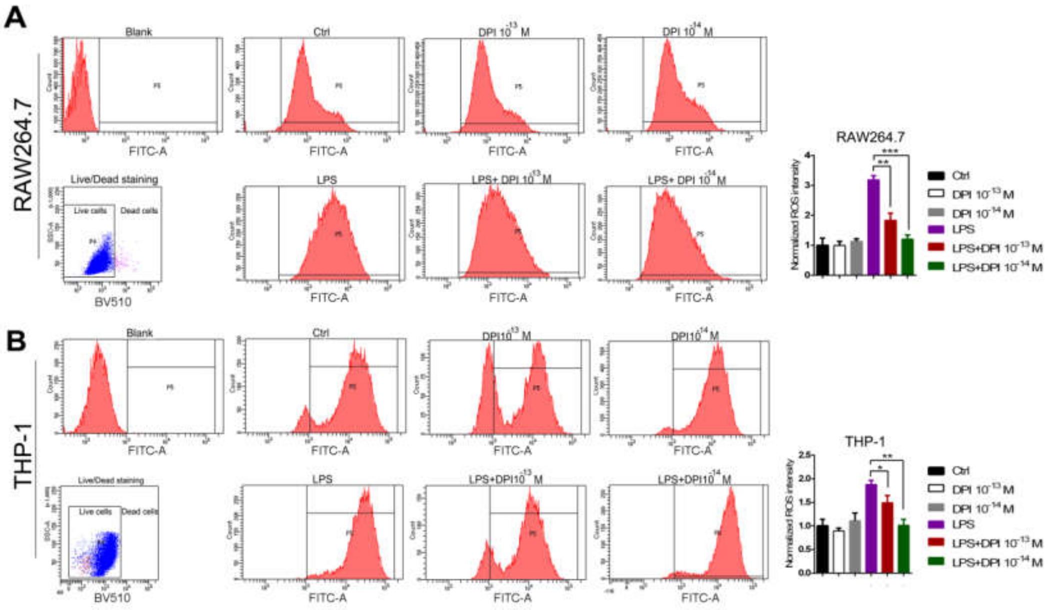

Supplementary Figure 3. An ultralow dose of DPI could inhibit ROS levels in vitro. Intracellular ROS levels were examined by analysis of DCFH-DA fluorescence intensity. RAW264.7 (A) and THP-1 cells (B) were treated with LPS (500 ng/mL) and/or DPI (10^{-13} M or 10^{-14} M) for 12 h. *$P < 0.05$, **$P < 0.01$, ***$P < 0.001$.

Supplementary Figure 4

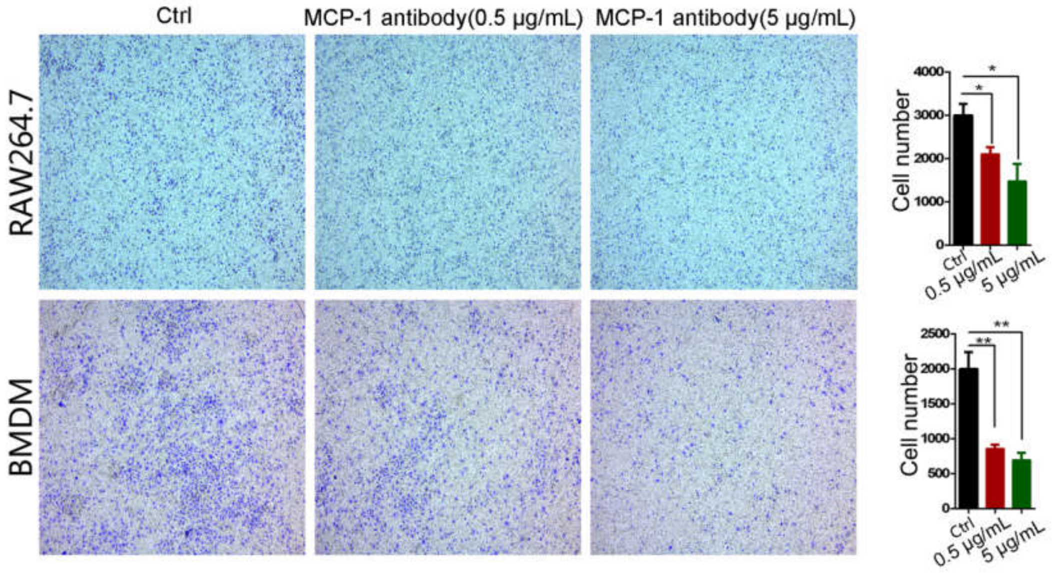

Supplementary Figure 4. MCP-1 antibody inhibits cell migration in vitro. RAW264.7 and BMDM cell migration, in the absence and presence of MCP-1 antibody, was assessed by transwell assay. Data are presented as the mean ± SEM of three separate experiments. *$P < 0.05$, **$P < 0.01$.

Supplementary Figure 5

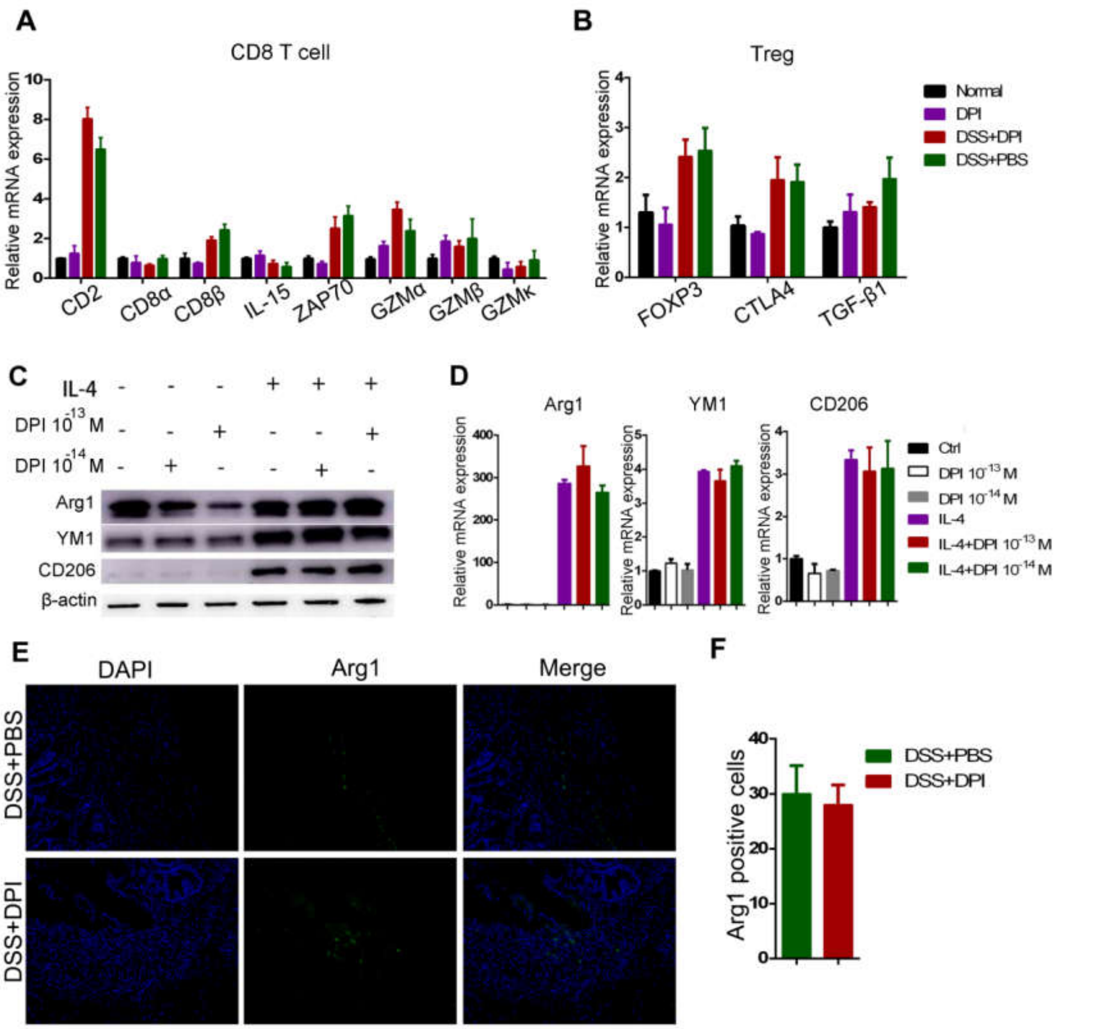

Supplementary Figure 5. An ultralow dose of DPI did not affect the activation of CD8$^+$ T cells, Tregs, or M2 macrophages. (A) Analysis of the relative gene expression associated with CD8$^+$ T cells and (B) Treg cells of the colonic lamina propria of mice on day 15 after DSS treatment (n = 7 per group). The gene expression levels were normalized to that of the Tbp gene for each sample. Analysis of M2-associated (C) protein expression and (D)

mRNA expression in BMDMs. Cells were treated with IL-4 and DPI (10^{-13} M or 10^{-14} M) for 12 h. (E) The M2 marker Arg1 was detected by immunofluorescence and (F) the number of $Arg1^{+}$ cells/ visual field in the colonic tissues from mice treated with DSS for 15 days was enumerated.

Supplementary Figure 6

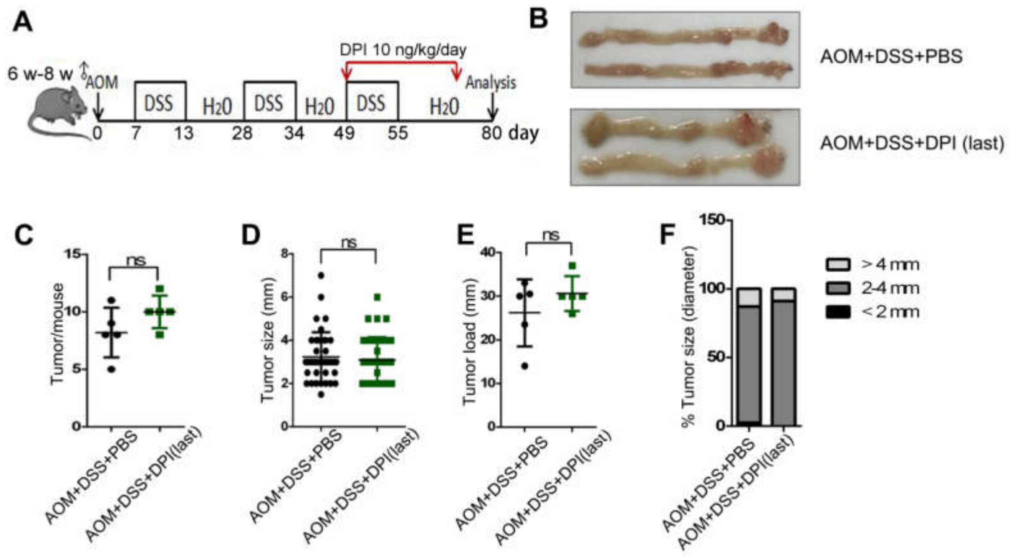

Supplementary Figure 6. There was no significant difference when DPI was used in the last round of DSS water treatment. (A) Mode pattern of CAC according to the DPI injection time. (B) Representative photographs of murine colons. (C) Number of tumors per mouse. (D) Tumor size and (E) tumor load. (F) Tumor distribution was measured for each group (n = 5 per group).

Supplementary Figure 7

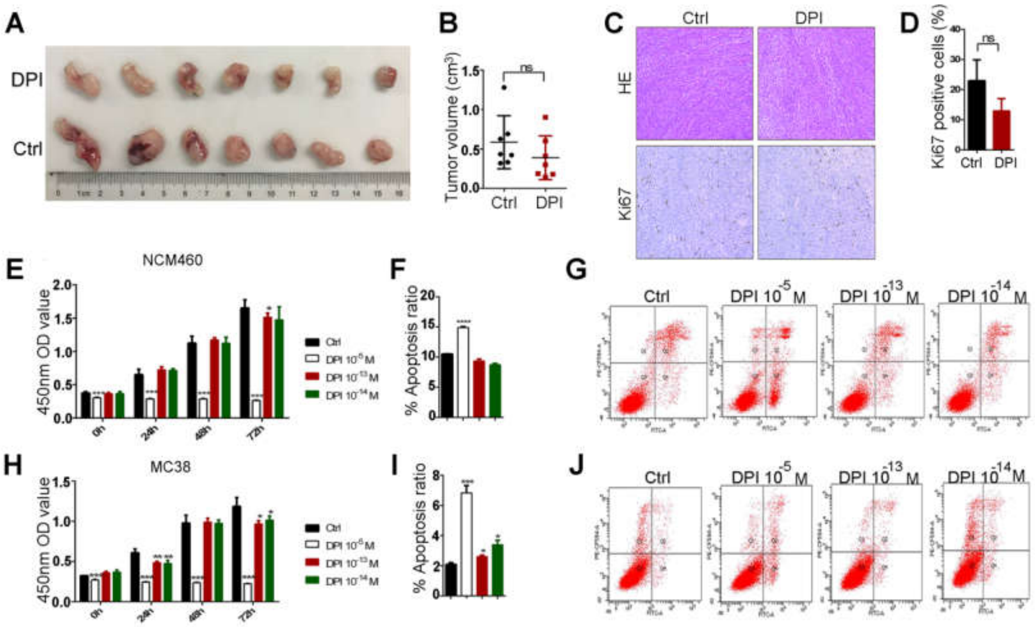

Supplementary Figure 7. An ultralow dose of DPI had no effect on the size of subcutaneous tumors. (A) Images of subcutaneous tumors from DPI - and PBS-treated mice (n = 7 per group). Tumors were analyzed on day 17 after tumor cells inoculation. (B) Tumor volumes. (C) Tumors were subjected to H&E staining and immunohistochemical staining of Ki67. (D) The number of $Ki67^{+}$ cells/ visual field in subcutaneous tumors from mice was enumerated. The proliferation of NCM460 (E) and MC38 cells (H) was determined by CCK-8 assay. The apoptosis of ncm460 (F-G) and MC38 (I-J) cells was detected by flow cytometry. *$P < 0.05$, **$P < 0.01$, ***$P < 0.001$.

Supplementary Figure 8

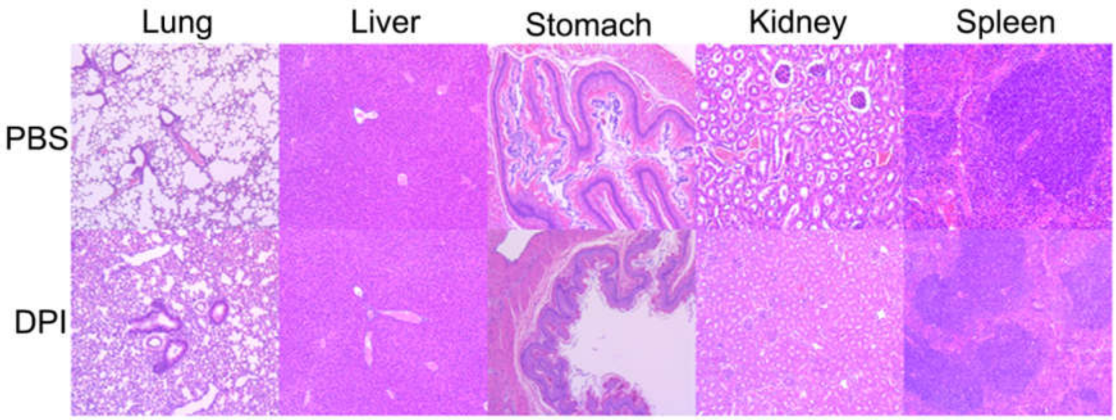

Supplementary Figure 8. An ultralow dose of DPI has no obvious systemic toxicity. Organs, including the lung, liver, stomach, kidney, and spleen, were dissected and stained with H&E.
